# Complete resection of pure anterior foramen magnum meningioma without neurovascular injuries poses a big challenge: Case report

**DOI:** 10.1016/j.amsu.2021.102265

**Published:** 2021-03-27

**Authors:** Ali Hammed, Moufid Mahfoud, Alaa Sulaiman, Adnan Najm, Salah Hammed

**Affiliations:** a Tishreen University Hospital, Department of Neurosurgery. Lattakia, Syria; b Faculty of Medicine. Aleppo, Syria

**Keywords:** Foramen magnum meningioma (FMM), Anterior FMM, Conservative transcondylar approach

## Abstract

**Introduction and importance:**

Meningiomas are common neoplasms representing 14.3–19% of all intracranial tumors. Among all the meningiomas, only 1.8–3.2% arises at the foramen magnum (FM) level.

Most of the lesions (68%–98%) arising anterolaterally, followed by postolateral, purely posterior and, more rarely, purely anterior.

**Case presentation:**

We report a case of A 42-year-old female presented with a history of neck pain with progressive spastic hemiparesis.

**Clinical Discussion:**

MRI revealed well-defied pure anterior and on both sides of vertebral artery, foramen magnum lesion. Through conservative transcondylar approach, Lesion was removed totally in a piecemeal fashion. Histopathology revealed meningothelial meningioma. The patient had a dramatic recovery.

**Conclusion:**

The exposure allowed by the far-lateral retrocondylar or partial transcondylar approach is adequate for resecting even anterior intradural FMMs.

Reports about Foramen magnum meningioma aren't common, but reports on pure anterior foramen magnum meningioma are very rare. The prerequisite for treating FM meningiomas (FMMs) is the perfect knowledge of the surgical anatomy. The opportunity to give the patient a symptom-free and normal life should not be missed in such cases.

## Highlights

• Only 1.8–3.2% of all meningiomas are located at foramen magnum (FM).
• Foramen magnum meningiomas (FMMs) represent a common histological tumor in a rare and eloquent location.
• FMMs are one of the most surgically complex conditions in neuro-oncologic surgery.
• The transcondylar approach is a more advanced technique and the total removal of ventral lesion could be achieved.

## 1 Introduction

Meningiomas are common neoplasms representing 14.3–19% of all intracranial tumors. Among all the meningiomas, only 1.8–3.2% arises at the foramen magnum (FM) level [1].

Most of the time, these are strictly intradural. Ten percent have an extradural extension: Most are intra- and extradural, and a few may be entirely extradural [[2], [3], [4]].

According to Boulton and Cusimano, the spinal dentate ligament delineate the anterior an posterior compartments with most of the lesions (68%–98%) arising anterolaterally, followed by postolateral, purely posterior and, more rarely, purely anterior [5].

FMMs are typically slow growing with an indolent course, but when they become symptomatic, they most commonly present with quadriparesis, sensory abnormalities, ataxia, and dysfunction of cranial nerves (CN) IX,X and XI [6].

FM contains several critical neuroanatomical and vascular structures. The neural structures include the cerebellar tonsils, inferior vermis, fourth ventricle, caudal aspect of the medulla, lower cranial nerves (CNs) (IX–XII), rostral aspect of the spinal cord, and upper cervical nerves (C-1 and C-2).

The exposure allowed by the far-lateral retrocondylar or partial transcondylar approach is adequate for resecting even anterior intradural FMMs. The prerequisite for treating FM meningiomas (FMMs) is the perfect knowledge of the surgical anatomy.

This work has been reported in line with the SCARE criteria [18,19].

## 2 Case presentation

A 42-year-old female presented to our department via community referral, with a history of neck pain with progressive spastic hemiparesis for 3 months. She had recent worsening of her limb weakness with no bowel and bladder involvement. Other symptoms were dysphagia and frequent disturbed sleep during night. Preoperatively, her Clinical examination revealed paralysis of sternocleidomastoid muscle and trapezius muscle and she had muscle power Medical Research Council (MRC) grade 3 on the right side.

All her deep tendon reflexes were exaggerated with bilateral planter extensor.

There were no subcutaneous nodules, hypopigmented macules, or other stigmata of NF.

The patient's review of systems and additional medical history surgical, family, psychosocial and pharmacologic were unremarkable.

**Fig. 1:**
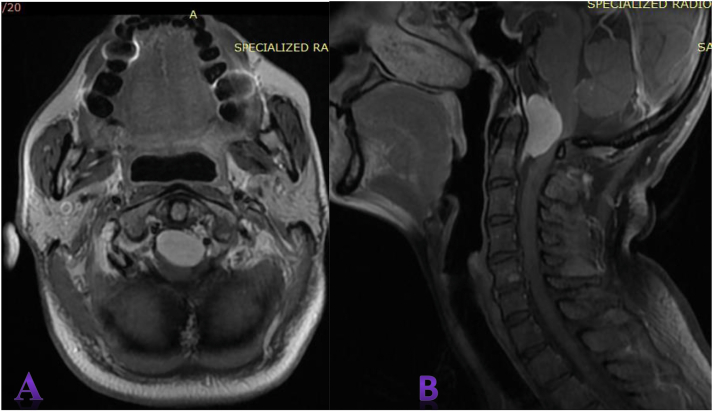
A: Post-contrast T1 weighted axial image, B: Post-contrast T1 weighted sagittal image: magneticresonance imaging (MRI) showing ventral placed, homogenously enhancing dural based lesion in the foramen magnum encasing both vertebral arteries suggestive of meningioma.

Magnetic resonance imaging (MRI) revealed well-defieddural-based mass lesion at foramen magnum, located anterior to neuroaxis and extending both up and down. causing severe compression of spinal cord. The cervicomedullary junction and upper cervical cord were sandwiched between the lesion and posterior border of foramen magnum (Fig. 1).

The differential diagnosis of a foramen magnum meningioma can include dermoids, epidermoids, teratomas, lipomas, hemangioblastomas, cavernous angiomas, giant thrombosed aneurysms of the vertebral artery, intramedullary cervical spinal cord tumors, and syringomyelia.

After obtaining the patient's informed consent, surgery was planned.

The procedure was done by a consultant neurosurgeon.

Through conservative transcondylar approach, approximately 10% of the posterior condyle was removed. The VA was identified and the posterior arch of C1 was resected. The tumor was decompressed preserving the neural and vascular structures. the lesion was removed totally in a piecemeal fashion. Simpson grade I was achieved.

Histopathology revealed a meningothelial meningioma (WHO-Grade 1).

Patient had a dramatic recovery from her symptoms postoperatively. After 4 months of follow up she showed marked improvement of power in her all limbs. MRC grade improves to 5 in all limbs which enable her to walk independently.

Routine follow-up by clinical observation and MRI was done.

**Fig. 2:**
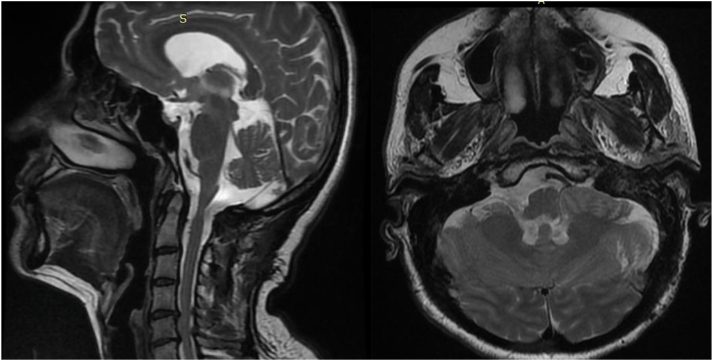
Post-operative T2 -MRI after 4 months shows no residual tumor.

Post-operative MRI after 4 months shows no residual tumors(Fig. 2).

## 3 Discussion

Meningiomas are extra-axial central nervous system (CNS) tumors that arise from the arachnoid cells of the dura mater. Meningiomas represent 14.3–19% of all intracranial tumors, the most common non-glial primary intracranial tumor [7].

A meningioma is considered to arise from the foramen magnum if the origin of the tumor is in the region bounded anteriorly by the lower third of the clivus and the upper edge of the body of C2, laterally by the jugular tubercles and the upper aspect of the C1 laminas, posteriorly by the anterior edge of the squamous occipital bone and C2 spinous process [8].

FMMs having the possibility to develop above, below, and on both sides of the VA. Meningiomas are more often located below the VA.

CN IX through CN XI arise as a series of rootlets along the anterior medulla, with the spinal component of the CN XI arising midway between the anterior and posterior spinal rootlets of the spinal cord.

Only 1.8–3.2% of all meningiomas are located at foramenmagnum (FM). Foramen magnum meningiomas (FMMs) represent a common histological tumor in a rare and eloquent location. As these tumors are indolent, there occurs a long interval between onset of symptoms and diagnosis [9].

The pathologic entity of FMM was first described in 1872. The first publication describing surgical removal was in 1922 [10].

The dorsolateral approach introduced by Koos et al., in1985 and later described by Perneczski in 1986, a member of the same school [11, 12], resembles the lateral suboccipital approach first described by Heros in 1985 [13]. Bothapproaches improved the exposure of the lower clivus and the upper cervical canal for removal of midline lesions, with minimal retraction of the cerebellum and complete sparing of the brain stem. Since then, posterolateral approaches, however named (dorsolateral tran scondylar or far lateral), have become a flexible corridor encompassing different degrees of craniocaudal and mediolateral exposure [14,15].

In general, lateral, far lateral and extreme lateral approaches have been recommended for anteriorly placed foramen magnum meningiomas [10,16].

The far-lateral transcondylar approach is a very useful approach to lesions located ventrolateral and even ventral to the brainstem and upper cervical spinal cord [17].

Due to the complexity of the region of the FM, the therapeutic and surgical strategy of FMM should be decided as per case. In our case, we reviewed the regional anatomy that helped us for a safe dissection of the neurovascular structures and aided in the avoidance of complications. Eventually, we achieved the favorable outcomes.

## 4 Conclusion

FMMs are one of the most surgically complex conditions in neuro-oncologic surgery, requiring special considerations due to the vicinity of the medulla oblongata, the lower cranial nerves, and the VA. There is no single best approach for FMMs and the optimal approach should be defied according to the localization and the extent of the tumor to minimize the extent of resultant morbidity.

The transcondylar approach is a more advanced technique than the far lateral approach And the total removal of ventral lesion could be achieved.

The opportunity to give the patient a symptom-free and normal life should not be missed in such cases.

## Sources of funding

This research did not receive any specific grant from funding agencies in the public, commercial, or not-for-profit sectors.

## Ethical approval

This study was not applicable for ethical approval.

## Consent

I have obtained written consent for publication of this case report from the patient and I can provide this should the Editor ask to see it.

## Author's contribution

Dr. Ali Hammed (corresponding author): Contribution to the paper: first author, data collection, data analysis and interpretation, writing the paper. PHD Dr. Moufid Mahfoud: Contribution to the paper:Main Surgeon. Treatment and examination of the patient. Writing Case Presentation. Dr. Salah Hammed: Contribution to the paper: Writing the paper. Dr. Alaa Sulaiman: Contribution to the paper: Writing the paper. Dr.Adnan Najm: Contribution to the paper: Writing the paper.

## Registration of research studies

The case report at hand is not a first-in-man case report of a novel technology or surgical technique, therefore a registration of these case reports according to Declaration of Helsinki 2013 is not required.

## Provenance and peer review

Not commissioned, externally peer-reviewed.

## Declaration of competing interest

All authors declared no conflict of interest.
